# Atrioventricular and sinus node dysfunction in mild acute COVID-19 disease: a case series

**DOI:** 10.1016/j.hroo.2024.05.005

**Published:** 2024-05-16

**Authors:** Andrea Surabova, Fu Guan, Florian Berger, Matthias Gass, Firat Duru, Thomas Wolber

**Affiliations:** ∗ Department of Cardiology, University Heart Center, University Hospital Zurich, Zurich, Switzerland; † Department of Cardiology, Children’s Hospital Zurich, Zurich, Switzerland; ‡ Department of Cardiology, Herz-Kreislauf-Praxis, Goetzis, Austria

**Keywords:** AV-block, Sinus node dysfunction, COVID-19, Syncope, Pacemaker

**Key Findings**

▪ Atrioventricular and sinus node dysfunction can be observed in acute COVID infection and may lead to syncope.
▪ In patients with symptoms suggesting bradyarrhythmia, electrocardiography monitoring should be performed.
▪ In patients without pre-existing cardiac conditions, sinus node dysfunction and atrioventricular conduction disturbances appear to be associated with a benign course and have no impact on long-term outcomes.
▪ Placement of a permanent pacemaker should be deferred in these patients, as spontaneous remission is likely to occur.

Various symptoms and complications have been described in association with SARS-CoV-2 infections in patients with and without pre-existing cardiovascular disease, including myocarditis, acute heart failure, and acute coronary syndrome. Cardiac arrhythmias have been noted in various viral infections, including those caused by SARS-CoV-2. Whether long-term consequences may arise from cardiac arrhythmias associated with acute COVID-19 infections is yet to be determined. We present data of 8 patients without prior cardiac disease who developed atrioventricular (AV) and sinus node disturbances during acute COVID-19 infection.

In this retrospective study, patients with a diagnosis of COVID-19, as confirmed by polymerase chain reaction testing, were included between January 2020 and June 2023. Patients were identified by chart review. The study was conducted in accordance with the regulations of the institutional and regional ethics committee. All patients provided written informed consent. All patients showed a mild clinical course of the infection yet experienced AV and sinus node dysfunction. Only patients without a history of cardiac disease and no prior conduction abnormalities were included. Three hospitalized patients were included, whereas 5 patients were treated on an outpatient basis. Patients requiring intensive care treatment, ventilation, or antiviral therapy were excluded. From 337 patients who were treated as outpatients for SARS-CoV-2 infections during the study period, 38 presenting with symptoms compatible with bradyarrhythmia received Holter electrocardiography (ECG) monitoring for up to 10 days. Of those, 5 patients with AV and sinus node dysfunction were included in the study. Echocardiography was performed within 24 hours of presentation in all patients. In hospitalized patients, cardiac magnetic resonance imaging (MRI) with gadolinium was performed during the hospital stay, whereas in outpatients, MRI was performed within 1 month after presentation. Outpatients provided a symptom protocol including blood pressure and body temperature measurements. A follow-up visit including blood testing was scheduled 5–8 days after the initial visit.

The symptoms and characteristics of all patients at admission are detailed in Table 1. Mean age was 54 ± 14 years and 75% were male. Hypertension was the sole common comorbidity, present in 50% of the patients. The mean left ventricular ejection fraction was 53 ± 6%. None of the patients had a history of cardiac symptoms or disease. Dizziness and malaise were the predominant symptoms upon presentation. Syncope with fracture of the temporal bone occurred in 1 patient. All hospitalized patients were continuously monitored with telemetry and outpatients received Holter monitoring, respectively. In 1 patient, a temporary pacemaker was inserted at admission due to recurrent 2:1 AV block, resulting in dizziness and nausea, and was removed on day 4. In all patients, arrhythmias were observed as recurrent episodes, rather than continuous block or pauses. The average duration of arrhythmias, defined as the time to full restoration of normal rhythm, was 2.63 ± 0.74 days. All patients exhibited second-degree AV block, while third-degree AV block was documented in half of the patients. The mean duration of the longest pauses observed in each patient was 3.6 ± 0.7 seconds, without any resulting syncope. Cardiac MRI imaging did not show signs of inflammation or scarring in any of the patients. None of the patients required intensive care or ventilation. None of the patients received antiviral treatment. The mean duration of ECG monitoring was 5.5 ± 1.2 days. None of the patients required permanent pacemaker implantation.

**Table 1:** Clinical parameters and observations

Age (y)	Sex	Treatment	HTN	LVEF (%)	Syncope	Monitoring (d)∗	Arrhythmia (d)†	AVB II	AVB III	SND	Maximum pause (s)‡
49	Male	Hospital	No	54	Yes	4	2	Yes	Yes	Yes	3
57	Female	Outpatient	No	46	No	7	2	Yes	Yes	Yes	4
45	Female	Outpatient	Yes	60	No	5	3	Yes	No	Yes	4
63	Male	Outpatient	No	47	No	6	4	Yes	Yes	No	4
56	Male	Outpatient	No	52	No	6	3	Yes	No	No	3
69	Male	Hospital	Yes	45	No	5	2	Yes	No	No	3
25	Male	Outpatient	Yes	57	No	7	3	Yes	Yes	Yes	5
66	Male	Hospital	Yes	60	No	4	2	Yes	No	Yes	3

AVB II = second-degree atrioventricular block; AVB III = third-degree atrioventricular block; HTN = hypertension; LVEF = left ventricular ejection fraction; SND = sinus node dysfunction.

∗ Length of telemetry/Holter monitoring.

† Duration of arrhythmia on electrocardiography monitoring.

‡ Duration of the longest pause observed.

During the follow-up period of 30 ± 7.5 months, all patients remained asymptomatic without any signs of arrhythmia or autonomic disbalance. Twelve-lead office ECGs were normal in all patients; however, Holter ECGs were not obtained.

Various pathophysiological mechanisms have been proposed for the development of arrhythmias in COVID-19, suggesting a multifactorial process. Binding to myocardial angiotensin-converting enzyme 2 receptors can induce myocarditis through direct cytotoxicity, which can cause arrhythmias.^1^ Immunohistochemically, SARS-CoV-2 spike and nucleocapsid antigens and viral genes have been detected in the AV node.^2^

Orthostatic tachycardia and intolerance have been observed during the acute phase of COVID infection and there is increasing recognition of postural orthostatic tachycardia syndrome and inappropriate sinus tachycardia in the context of long COVID. Histopathologic studies have demonstrated that the presence of SARS-CoV-2 RNA in the vagus nerve as well as vagus nerve inflammation are widespread in COVID-19.^3^ Furthermore, autoantibodies against beta receptors and cholinergic muscarinic receptors as a cause for autonomic imbalance have been discussed.^4^

Several case reports and series have been published from patients with SARS-CoV-2–related bradyarrhythmia and conduction disturbances. The incidence and significance of new onset AV block has been discussed controversially. In a study of 200 hospitalized COVID patients,^5^ including patients with pre-existing coronary disease (23%), atrial fibrillation (18%), diabetes (38%), and chronic kidney disease (16%), the incidence of new onset AV block was 5.5%. Nine out of these 11 patients were reported to have first-degree AV block and were managed conservatively, in 2 patients a pacemaker was placed, and 3 patients died.

A study examining device implants in 2 institutions between March 2020 and February 2021 showed that the prevalence of bradyarrhythmias needing pacing remained unchanged during the pandemic, and all patients in this study required permanent pacing.^6^

In the current study of conduction disturbances in the context of mild SARS-CoV-2 infection, the estimated incidence of AV block in outpatients without pre-existing cardiac disease was 1.5%. These patients primarily exhibited bradycardia, attributed to sinus nodal dysfunction, and AV block, which were frequently associated with dizziness. No additional signs or symptoms of autonomic dysfunction, such as orthostatic hypotension, gastrointestinal disturbances, abnormal sweating patterns, or urinary symptoms, were observed. This might indicate a reversible, direct impact of COVID-19 on the myocardium and cardiac conduction, rather than on autonomic nervous function. In all reported patients, AV and sinus node dysfunction resolved within 2–3 days of presentation and remained normal thereafter. None of the patients reported any symptoms of bradycardia or autonomic dysfunction during follow-up.

While in severe SARS-CoV-2 infections arrhythmias may be an indicator of increased risk and adverse outcome, in patients with mild COVID-19 disease and no pre-existing cardiac condition, sinus node dysfunction and AV conduction disturbances, even when resulting in syncope, appear to be associated with a benign course and have no impact on long-term outcomes. Symptomatic patients should be monitored for bradyarrhythmias; however, the placement of a permanent pacemaker should be deferred as spontaneous remission is likely to occur.

## References

[1] Mezache L., Nuovo G.J., Suster D. (2022). Histologic, viral, and molecular correlates of heart disease in fatal COVID-19. Ann Diagn Pathol.

[2] Jakovac H., Ferenčić A., Stemberger C., Mohar Vitezić B., Cuculić D. (2022). Detection of SARS-CoV-2 antigens in the AV-node of a cardiac conduction system-a case report. Trop Med Infect Dis.

[3] Woo M.S., Shafiq M., Fitzek A. (2023). Vagus nerve inflammation contributes to dysautonomia in COVID-19. Acta Neuropathol (Berl).

[4] Goldstein D.S. (2021). The possible association between COVID-19 and postural tachycardia syndrome. Heart Rhythm.

[5] Lao N., Lim J., Bashir H. (2022). Incidence of atrioventricular blocks and its association with in-hospital mortality and morbidity in patients with coronavirus disease 2019. J Cardiol.

[6] Akhtar Z., Leung L.W., Kontogiannis C. (2021). Prevalence of bradyarrhythmias needing pacing in COVID-19. Pacing Clin Electrophysiol.

